# Purification and immobilization of *β*-glucosidase using surface modified mesoporous silica Santa Barbara Amorphous 15 for eco-friendly preparation of sagittatoside A

**DOI:** 10.1007/s13659-024-00471-x

**Published:** 2024-08-23

**Authors:** Ya-Ya Yang, Shun-Li Jing, Jia-Li Shao, Ji-Xuan Chen, Wei-Feng Zhang, Si-Yuan Wan, Yu-Ping Shen, Huan Yang, Wei Yu

**Affiliations:** 1 School of Pharmacy, Jiangsu University, Zhenjiang, 212013 Jiangsu Province People’s Republic of China; 2 Development Department, Jiangsu Grand Xianle Pharmaceutical Co., Ltd, Yancheng, 224555 People’s Republic of China

**Keywords:** Immobilized *β-*glucosidase, Modified SBA-15, Sagittatoside A

## Abstract

Functionalized mesoporous materials have become a promising carrier for enzyme immobilization. In this study, Santa Barbara Amorphous 15 (SBA-15) was modified by N-aminoethyl-*γ*-aminopropyl trimethoxy (R). R-SBA-15 was employed to purify and immobilize recombinant *β*-glucosidase from *Terrabacter ginsenosidimutans* (BgpA) in one step for the first time. Optimum pH of the constructed R-SBA-15@BgpA were 7.0, and it has 20 ℃ higher optimal temperature than free enzyme. Relative activity of R-SBA-15@BgpA still retained > 70% at 42 ℃ after 8-h incubation. The investigation on organic reagent resistance revealed that the immobilized enzyme can maintain strong stability in 15% DMSO. In leaching test and evaluation of storage stability, only trace amount of protein was detected in buffer of the immobilized enzyme after storage at 4 ℃ for 33 days, and the immobilized BgpA still maintained > 50% relative activity. It also demonstrated good reusability, with 76.1% relative activity remaining after fourteen successive enzymatic hydrolyses of epimedin A to sagittatoside A. The newly proposed strategy is an effective approach for the purification and immobilization of BgpA concurrently. In addition, R-SBA-15@BgpA was demonstrated to have high efficiency and stability in this application, suggesting its great feasibility and potential to produce bioactive compounds such as secondary glycosides or aglycones from natural products.

**Graphical Abstract:**

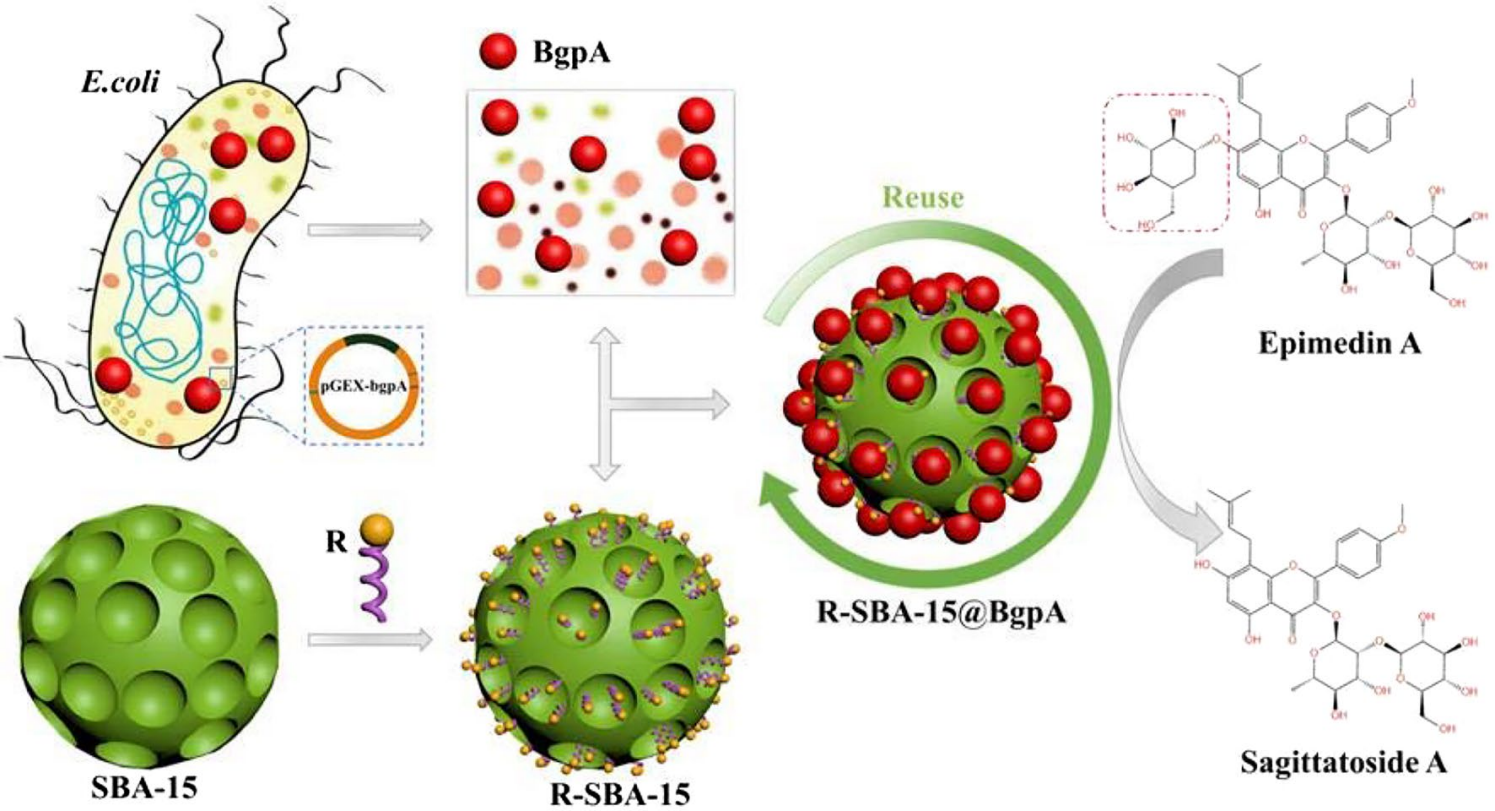

## Introduction

Enzyme immobilization is an essential technology for commercializing biocatalysis in recent decades. It imparts desired performance in stability, recoverability, and reusability, which can much improve the effectiveness and eco-friendliness of biocatalysts [1]. This technology has been widely used in the biological industry, medical and clinical diagnosis, chemical analysis, and environmental protection and is playing a significant role in such applications [2–5]. Recent advances in enzyme immobilization have strongly highlighted its advantages, and plenty of new techniques for enzyme immobilization have emerged. For instance, immobilized enzyme catalysis and photocatalysis was cascaded in a continuous-microflow manner to realize online reuse of trypsin, eliminate cross-inhibitions between two-step reaction processes, provide stronger and more uniform illumination, and enhance process efficiency [6]. In addition, a novel strategy was well-established in our previous investigation, which harnessed *α*-glucosidase functionalized magnetic beads to fish out potential *α*-glucosidase inhibitors from *Epimedii Folium* extract. This approach has a vital prospect in conveniently screening bioactive compounds that target various receptors, which provide an efficient platform for new drug development from natural products [7].

Appropriate carrier is a crucial component during the preparation of immobilized enzymes. Carriers differ in their physio-chemical properties, which directly affect their interaction with enzymes, moreover enzyme activity. Therefore, the innovation and preparation of novel carriers for enzyme immobilization have become a hot topic nowadays. In these decades, a great of attention has been paid to the controlled immobilization of enzyme on carbon nanotubes (CNTs). Through introduction of the functional groups, enzyme can be specifically and precisely bound onto CNTs [8]. Metal–organic frameworks (MOFs) are widely used in the fields of gas adsorption separation, biocatalysis and energy storage. For example, UiO-66, a typical MOF, has been discovered to be effective in immobilizing *Aspergillus niger* lipase. In the catalysis of lipase-mediated biodiesel production, the immobilized lipase performed at a much faster reaction rate and produced a significantly higher yield than its non-immobilized lipase [9]. However, enzymes immobilization on MOFs faces some challenges, such as low loading rate of enzymes, meanwhile it is difficult to ensure the original activity of enzymes [10]. In addition, Chitosan is a natural amino polysaccharide with good mechanical properties, easy modification, and low cost. The presence of amino and hydroxyl groups in chitosan allows for a variety of immobilization methods for enzymes [11–13]. For instance, magnetic chitosan (MCH) microspheres were synthesized via phase transformation and the grafting of polydopamine (PDA), followed by directly immobilizing lipase through Schiff's base reaction. The formed MCH@PDA-lipase showed better thermal and storage stability at different temperatures than free lipase [14]. Kim et al. [15] explored the potential application of chitosan nonwoven fabric immobilized with enzymes in biomedical textiles. The trypsin was immobilized on the glutaraldehyde-crosslinked chitosan nonwoven fabric, and it was found that immobilized trypsin had weaker pH stability but stronger thermal stability than free trypsin. Immobilized trypsin retained 50% of its initial activity after being consecutively used fifteen times, and it still maintained 80% of its initial activity after storage at 4 °C for 20 days. However, chitosan also has several limitations. As a carrier, chitosan gel has poor liquid flowability, making long-term continuous operations difficultly. Additionally, the use of glutaraldehyde in conjunction with chitosan can largely lead to enzyme deactivation [16].

The most common inorganic carriers are silicates [17]. Among them, mesoporous materials are widely recognized for their properties such as high specific surface area, uniform pore distribution, tunable pore size, and excellent thermal stability. These advantageous features make such materials ideal candidates for enzyme immobilization [18, 19]. Santa Barbara Amorphous 15 (SBA-15), as a representative of mesoporous materials, was extensively used in lipase immobilization due to its excellent properties, such as large specific area, proper pore diameter, and great possibility of surface modification [20–27]. In addition, the tin-modified SBA-15 has a high specific activity, while in the leaching tests, no desorption lipase was detected in the buffer, indicating a strong interaction between the lipase and the modified silica [26]. For example, SBA-15 was modified with 3-chloropropyltriethoxysilane, and the lipase immobilized on SBA-15-Cl had higher thermal stability, pH stability, and storage stability than free lipase. It could be reused up to eight times without a significant decrease in enzyme activity. In addition, due to the decreased pore size of SBA-15-Cl, enzyme leakage was significantly improved [28].

Starting from the perspective of new drug development, after long-term efforts, researchers have isolated many effective ingredients from natural medicines [29]. Through pharmacological metabolism investigation, it has been well-recognized that primary glycosides are converted into their secondary forms in gastrointestinal tract of human body to exert their curative effects [30, 31]. Therefore, preparation of secondary glycosides by enzymatic hydrolysis has become a hot research field. Recombinant enzyme is the most preferred glycoside hydrolase that plays an important role in the hydrolysis of natural products such as icariin, epimedin A, and ginsenosides. Recombinant glucosidases are frequently used to hydrolyze the glycosidic bonds of active components in medicinal plants. For instance, an *β*-glucosidase (71.14 kDa) cloned from *Terrabacter ginsenosidimutans* Gsoil 3082T (BgpA) demonstrated a hydrolytic activity toward sugar moieties at C6 and C20 positions in the protopanaxatriol-type ginsenosides. It could hydrolyze ginsenosides Re and Rg1 to 20(S)-protopanaxatriol aglycone through the formation of ginsenoside while the optimum temperature was 37 °C. Therefore, BgpA can be used to effectively produce rare ginsenosides with high biological activity [32, 33]. Recombinant glycosidase has the characteristics of high catalytic efficiency, high specificity and mild reaction conditions. Sagittatoside A is of very low content in Epimedium and it is a secondary flavonol glycoside by specific hydrolysis of -OR_2_ on epimedin A. This natural product could be readily transported into plasma and play an important bioactive role in vivo [34]. According to the published literature, glycoside hydrolase is the most commonly used enzyme for epimedin A hydrolysis [35]. However, there are numerous limitations of free glycosidases, including high costs associated with preparation and purification, difficulty in recycling of enzyme, and poor thermal stability. To select an appropriate immobilization method is of great significance for improving the stability and activity of glucosidase and promoting its large-scale industrial application.

**Scheme 1:**
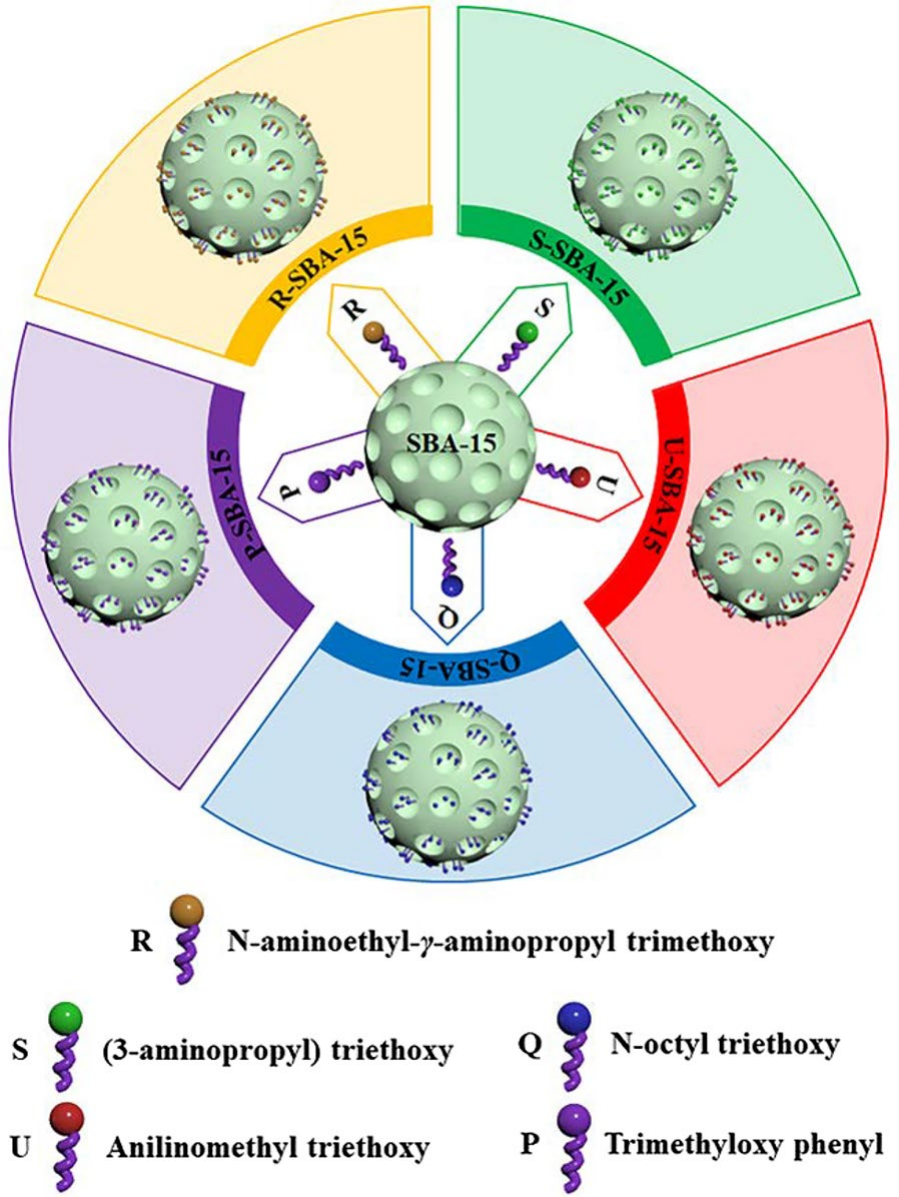
Surface Modification of SBA-15. *SBA-15* Santa Barbara Amorphous 15, *X-SBA-15* different group modified SBA-15, *R-SBA-15* N-aminoethyl-*γ*-aminopropyl trimethoxy modified SBA-15, *S-SBA-15* (3-Aminopropyl) triethoxy modified SBA-15, *P-SBA-15* Trimethyloxy phenyl modified SBA-15, *Q-SBA-15* N-octyl triethoxy modified SBA-15, *U-SBA-15* Anilinomethyl triethoxy modified SBA-15

In this study, recombinant BgpA was purified and immobilized on N-aminoethyl-*γ*-aminopropyl trimethoxy modified SBA-15 (R-SBA-15) in one step for the first time (Scheme 1), and the effect of immobilization conditions was investigated by one-factor-at-a-time (OFAT) experiment. The resulting immobilized BgpA was evaluated for thermal stability, organic solvent resistance, storage stability, and its reusability for conversion of Epimedin A to prepare Sagittatoside A was also assessed. This work intended to propose an efficient approach for enzyme immobilization, meanwhile it has shown great feasibility and potential to provide a laboratory-scale experimental foundation for eco-friendly preparation of natural bioactive compounds in industrial application.

## Results and discussion

### Production of BgpA

**Fig. 1:**
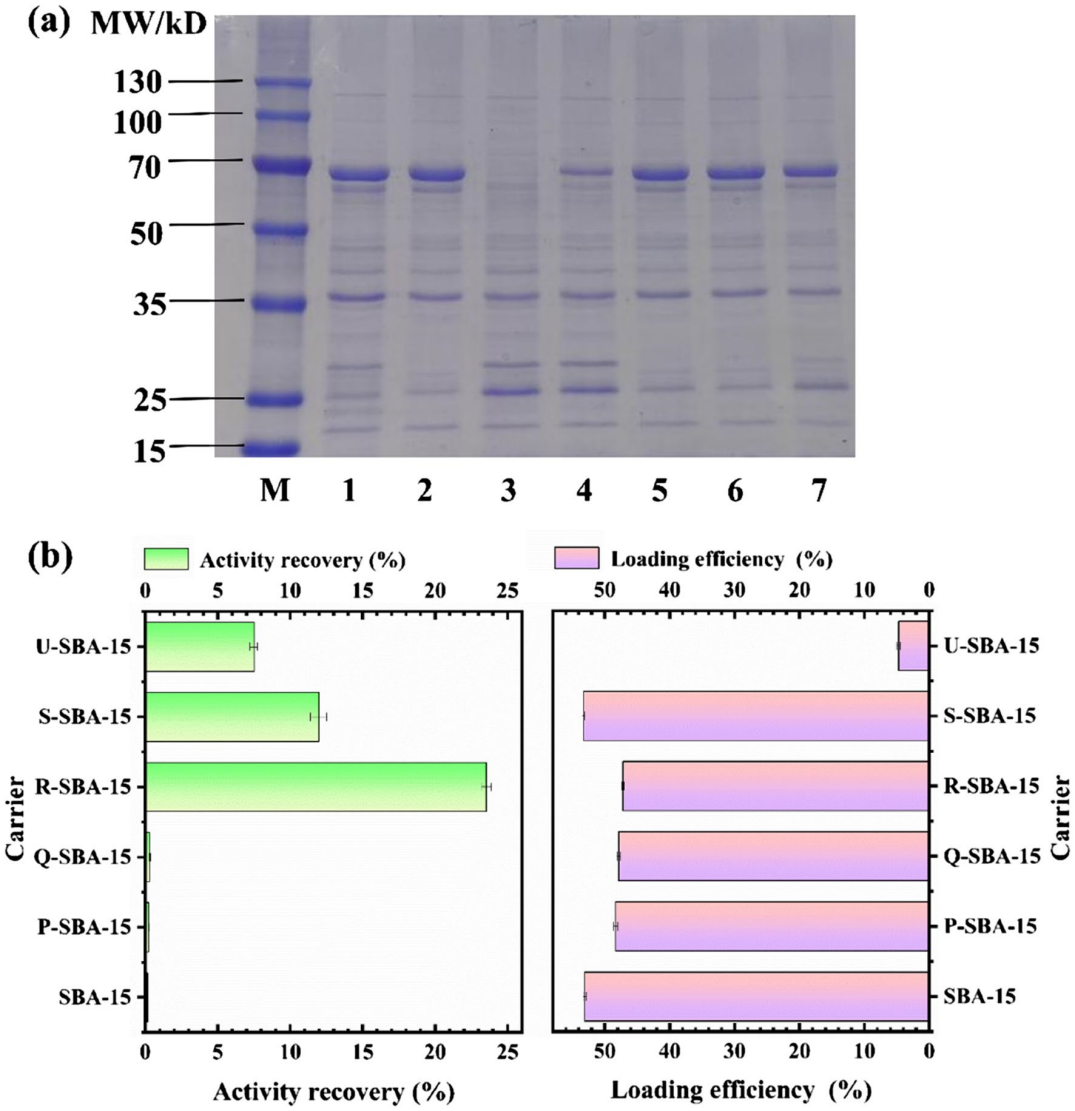
Immobilization of BgpA onto SBA-15 or X-SBA-15. **a** SDS-PAGE profiles. Lane L: protein marker; Lane 1: crude BgpA; Lane 2: unbound proteins of SBA-15; Lane 3: unbound proteins of R-SBA-15; Lane 4: unbound proteins of S-SBA-15; Lane 5: unbound proteins of P-SBA-15; Lane 6: unbound proteins of Q-SBA-15; Lane 7: unbound proteins of U-SBA-15. **b** activity recovery and loading efficiency of SBA-15 or X-SBA-15. *BgpA* recombinant *β*-glucosidase from *Terrabacter ginsenosidimutans*, *SBA-15* Santa Barbara Amorphous 15, *X-SBA-15* different group modified SBA-15, *R-SBA-15* N-aminoethyl-*γ*-aminopropyl trimethoxy modified SBA-15, *S-SBA-15* (3-Aminopropyl) triethoxy modified SBA-15, *P-SBA-15* Trimethyloxy phenyl modified SBA-15, *Q-SBA-15* N-octyl triethoxy modified SBA-15, *U-SBA-15* Anilinomethyl triethoxy modified SBA-15

GST-BgpA gene was expressed in *E. coli* under the control of the IPTG-inducible promoter Ptac. Induction with 0.5 mM IPTG at 20 ℃ for 6 h produced the maximum level of soluble active enzyme, resulting in 1,000 mL of crude enzyme solution. The protein concentration was 3.886 ± 0.116 mg/mL and specific activity of BgpA in crude enzyme solution was 82.988 ± 3.581 U/mg. The results of SDS-PAGE were shown in Fig. 1a (Lane 1) and the size of the target protein band at *ca.* 70 kDa were in good agreement with the estimated size based on the translated polypeptide sequence (71.14 kDa) [32], which suggested that the glucosidase was physiologically active as a trimeric protein. However, some protein impurities were also observed, which originated from endogenous proteins of *E. coli*. and need to be removed by conventional column chromatography and precipitation. In this study, recombinant BgpA was purified and immobilized from the crude enzyme in one step by using the mesoporous silica SBA-15 after surface modification, so there was no extra step to remove protein impurities in crude enzyme by routine operations, and the removal of protein impurities was achieved at the time of immobilization of BgpA on R-SBA-15.

### Immobilization of BgpA onto the carriers

To achieve the highest activity recovery, a total of six different carriers, namely SBA-15, Anilinomethyl triethoxy, (3-Aminopropyl) triethoxy, N-aminoethyl-γ-aminopropyl trimethoxy, N-octyl triethoxy or Trimethyloxy phenyl modified SBA-15 (U-SBA-15, S-SBA-15, R-SBA-15, Q-SBA-15 or P-SBA-15) were compared for BgpA immobilization. The characteristics of the carrier could affect the properties of the immobilized enzyme. For the X-SBA-15 carriers, different performance in loading efficiency and activity recovery was observed when an equal amount of BgpA was added. As shown in Fig. 1b, the activity recovery of U-SBA-15@BgpA, P-SBA-15@BgpA and Q-SBA-15@BgpA was all below 10%, not much higher than SBA-15@BgpA. Meanwhile, except for U-SBA-15 exhibiting an extremely low loading efficiency at *ca.* 5%, the efficiency of the other five carriers were around 50%. In addition, S-SBA-15@BgpA exhibited an activity recovery above 10% and the highest loading efficiency among those six carriers, while R-SBA-15@BgpA showed a significant advantage with activity recovery of 23.5%. S-SBA-15 and R-SBA-15 demonstrated their good performance for immobilizing BgpA and it implied that the enzyme has strong affinity to some specific carriers. The similarity between these two carriers was that they both carry amino groups on their functionalized surfaces, which could be responsible for the high activity recovery of S-SBA-15@BgpA and R-SBA-15@BgpA.

Interestingly, seen from the results of SDS-PAGE in Fig. 1a, both R-SBA-15 (Lane 3) and S-SBA-15 (Lane 4) among the six carriers were found to specifically immobilize target protein BgpA (*ca.* 70 kDa), meanwhile other protein bands did not significantly vary. Especially, the recombinant BgpA in the crude enzyme was completely captured by R-SBA-15 to achieve immobilization and purification of BgpA in one step, demonstrating a specific interaction between the carrier and the enzyme. The rationale for this finding could be that organic modification has changed the micro-environment of original SBA-15, and different organic functional groups led to differences in the micro-environment, which could potentially alter the characteristics of the immobilization agent [36]. In this application, the modification by N-aminoethyl-γ-aminopropyl trimethoxy on SBA-15 has achieved better specific affinity with BgpA than (3-Aminopropyl) triethoxy, Trimethyloxy phenyl, N-octyl triethoxy, and Anilinomethyl triethoxy. After recombinant BgpA has been adsorbed onto R-SBA-15, the amount of protonated species becomes dominant as a result of the formation of -NH_3_^+^•••^−^OOC- as a result of the ionic interaction between surface amine groups and carboxyl groups of BgpA [37].

### Optimization of R-SBA-15@BgpA preparation conditions

**Fig. 2:**
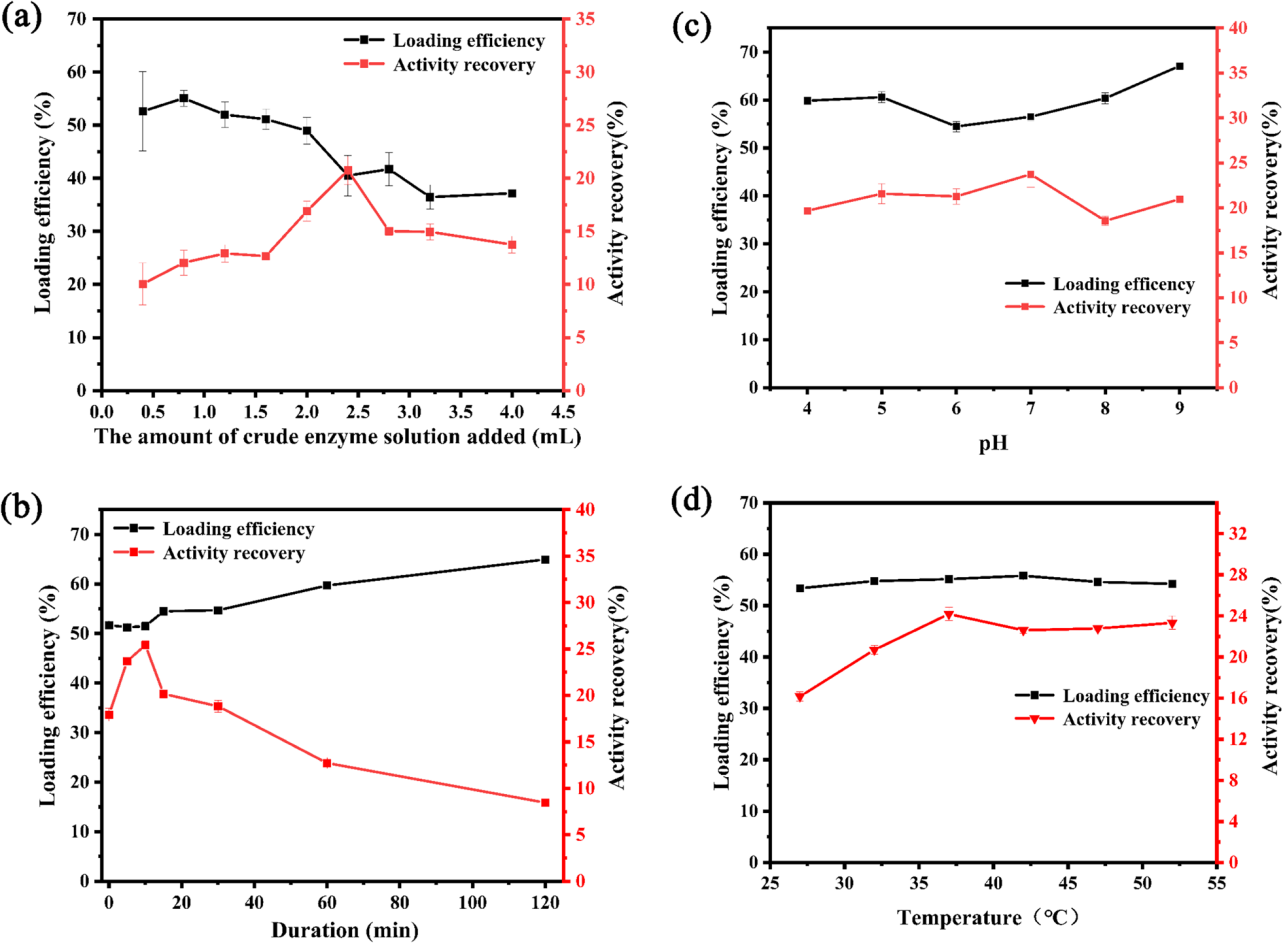
Effect of the **a** the amount of crude enzyme solution added, **b** adsorption duration, **c** pH and **d** temperature on the activity recovery and loading efficiency of R-SBA-15@BgpA. *R-SBA-15@BgpA* immobilized BgpA on N-aminoethyl-*γ*-aminopropyl trimethoxy group modified Santa Barbara Amorphous 15

There are several key factors affecting immobilization were studied by one-factor-at-a-time experiments to achieve the best performance of R-SBA-15@BgpA. It was seen from Fig. 2 that the amount of crude enzyme solution added, adsorption duration, pH of buffer solution, and temperature have differently affected loading efficiency and activity recovery, respectively.

As shown in Fig. 2a, the loading efficiency was at the highest level when 0.8 mL of crude enzyme solution was added, and the activity recovery continued to increase as more enzyme solution was added. When the enzyme solution volume reached 2.4 mL, the activity recovery arrived at its maximum. However, with further addition of enzyme solution, both the activity recovery and loading efficiency showed a decreasing trend. This could be due to saturated protein immobilization on the carrier. In addition, the enzyme became oversaturated on the carrier, probably masking the active sites of the enzyme immobilized on the carrier.

Seen from Fig. 2b, the loading efficiency had already reached 50% after the enzyme solution was poured into the carrier prior to the mixing and sonication for 1 min, indicating an extremely fast immobilization of BgpA on R-SBA-15. As the adsorption lasted for a longer time, the maximum activity recovery was achieved 25.4% at 15 min. Then the loading efficiency still slowly increased to 64.9% even if the activity recovery kept decreasing. High protein loading created steric hindrance between protein molecules, retarding the diffusion of substrates and products; or it led to a high degree of enzyme cross-linking, which could block the active sites on the enzyme and cause a decrease in the activity recovery.

**Fig. 3:**
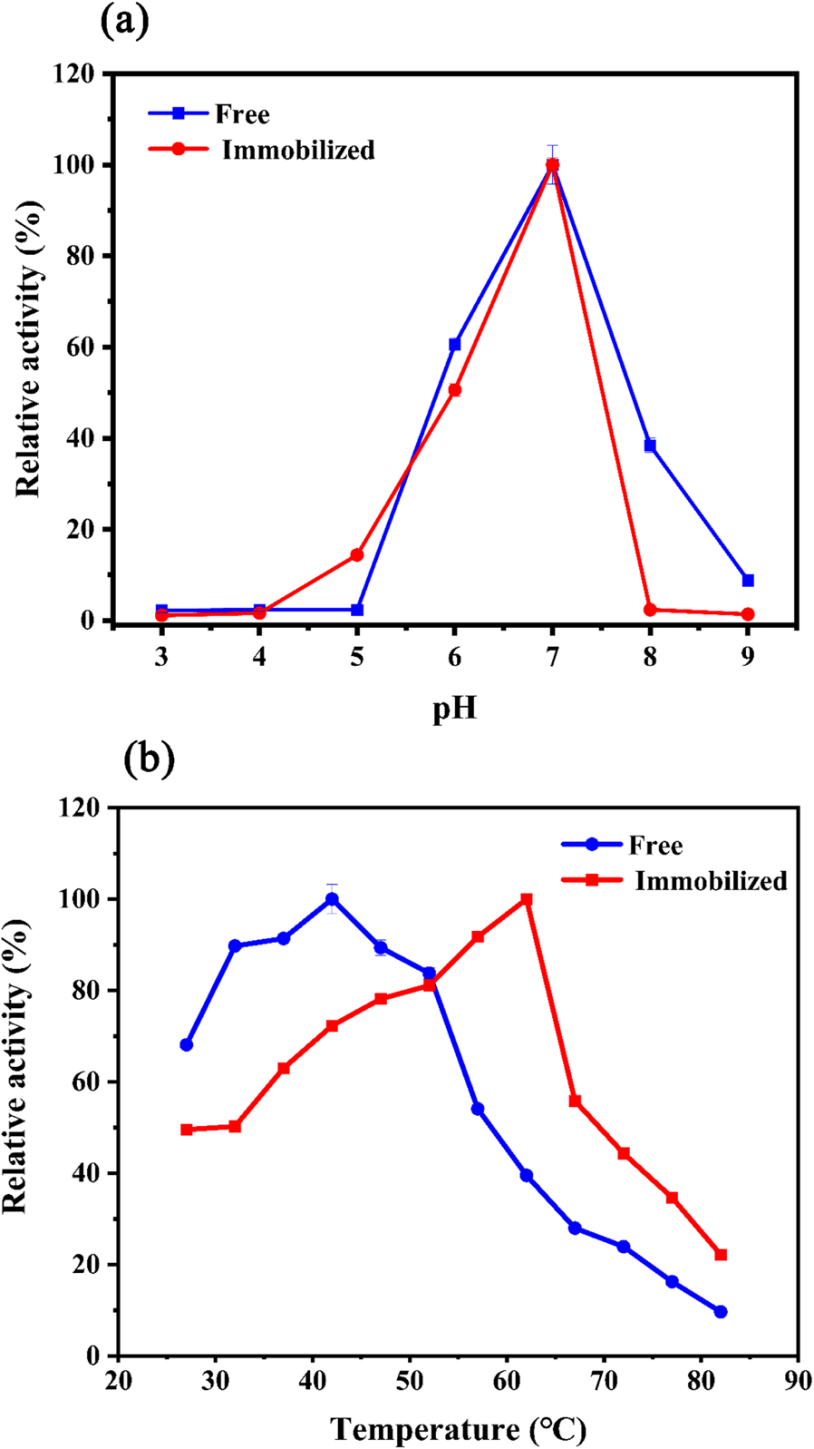
Effect of **a** pH and **b** temperature on the activity of the free BgpA and R-SBA-15@BgpA. *BgpA* recombinant *β*-glucosidase from *Terrabacter ginsenosidimutans*, *R-SBA-15@BgpA* immobilized BgpA on N-aminoethyl-*γ*-aminopropyl trimethoxy modified Santa Barbara Amorphous 15

As shown in Fig. 3c, the difference in the effects of pH on the activity recovery and loading efficiency of immobilized enzymes was not significant. The highest enzyme activity recovery was achieved when the pH was set at 7.0.

Seen from Fig. 3d, with the increase of temperature, the activity recovery of immobilized enzyme showed an increasing trend followed by a decreasing trend. There is no significant difference in the loading efficiency, and the optimal activity recovery 24.2% was achieved at 37℃. This suggested that the temperature could influence the distribution of enzyme molecules in the carrier, thus affecting the exposure of the active site.

### Optimum pH and temperature

Figure 3a showed the relative activities of free BgpA and the immobilized enzyme R-SBA-15@BgpA in buffers of different pH values ranging from 3.0 to 9.0. It can be observed that R-SBA-15@BgpA had the highest relative activity at pH 7.0, and showed no significant advantage over the free enzyme. This indicated that the carrier R-SBA-15 did not have a significant beneficial effect on the acid–base tolerance of BgpA. On the other hand, Fig. 3b showed the relative activities of free BgpA and the immobilized enzyme R-SBA-15@BgpA at different temperatures ranging from 27 to 82 °C. It can be seen that R-SBA-15@BgpA had a 20 °C higher optimal temperature than the free enzyme, which was increased from 42 to 62 °C.

### Thermal stability

**Fig. 4:**
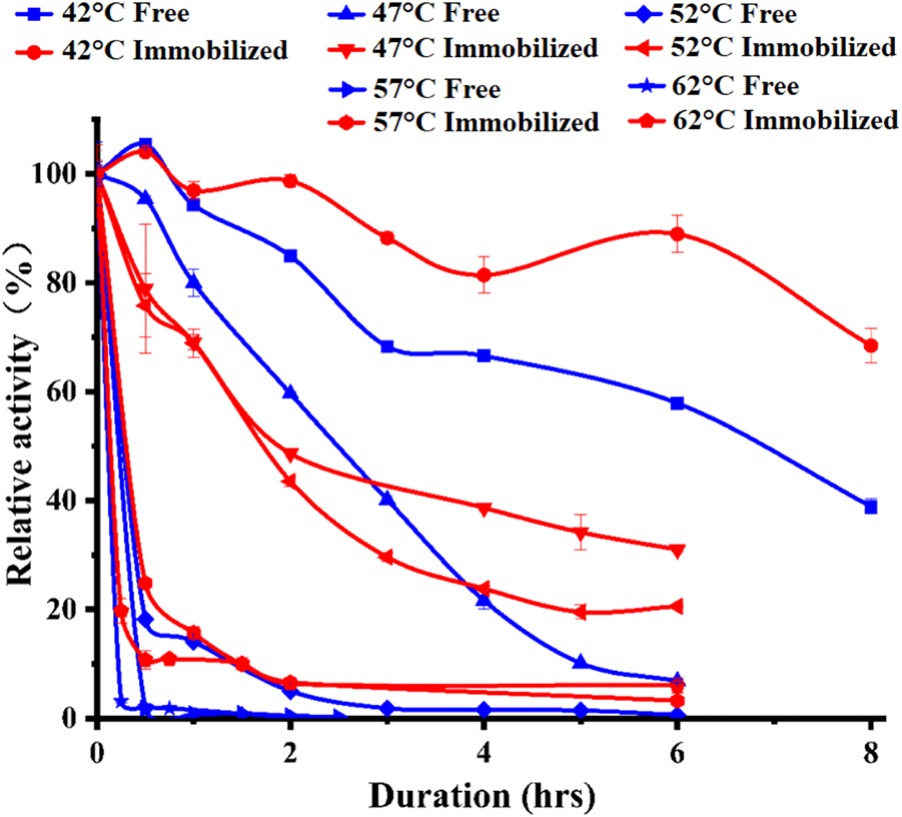
Thermal stability of free BgpA and R-SBA-15@BgpA at 42 ℃, 47 ℃, 52 ℃, 57 ℃, 62 ℃. *BgpA* recombinant *β*-glucosidase from *Terrabacter ginsenosidimutans*, *R-SBA-15@BgpA* immobilized BgpA on N-aminoethyl-*γ*-aminopropyl trimethoxy modified Santa Barbara Amorphous 15

To further confirm the advantage of the immobilized enzyme R-SBA-15@BgpA, thermal stability experiment was subsequently performed. The relative activity of immobilized and free BgpA at different temperatures after incubation was shown in Fig. 4. After continuous incubation at 42 °C for 8.0 h, immobilized BgpA still retained relative activity above 70%, while free enzyme lost 60% of its initial enzyme activity. However, after incubation at 47 °C for 6 h, immobilized enzyme retained one-third of its initial enzyme activity, while the activity of free enzyme was only about one-tenth. When the temperature was raised to 52 °C, the activity of free enzyme decreased sharply and the relative activity dropped to below 10% after 2-h incubation, while that of immobilized enzyme remained at around 40%. When the temperature continued to rise to 57 °C or above, the relative activity of both free and immobilized enzymes decreased in a rapid manner. Within one-hour incubation, the relative activity of immobilized enzyme decreased to *ca.* 10%, while the activity of free enzyme almost disappeared. The results confirm the role of immobilization in improving the thermal stability of BgpA. The good thermal stability could be attributed to the suitable microenvironment provided by the functionalized mesoporous silica, which prevented BgpA from thermal denaturation [36]. Strong thermal stability is a crucial feature, which could equip the enzyme with a good prospect in its industrial applications.

### Organic solvent resistance

**Fig. 5:**
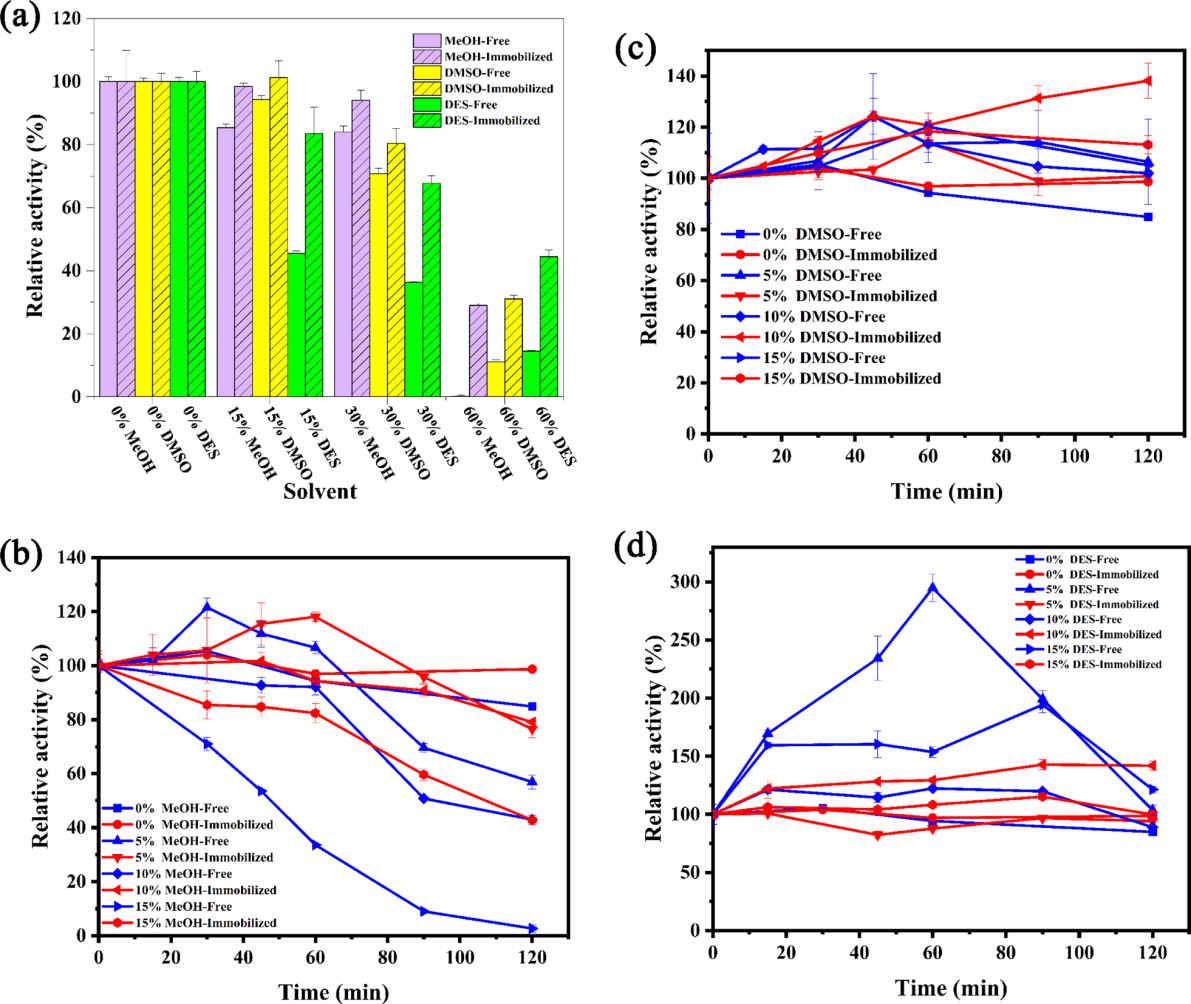
Organic solvents resistance of free BgpA and R-SBA-15@BgpA: **a** different proportions of different organic reagents; **b** different proportions of methanol; **c** different proportions of DMSO; **d** different proportions of DES. *BgpA* recombinant *β*-glucosidase from *Terrabacter ginsenosidimutans*, *R-SBA-15@BgpA* immobilized BgpA on N-aminoethyl-*γ*-aminopropyl trimethoxy modified Santa Barbara Amorphous 15, *DES* Deep eutectic solvent

The relative activity of free and immobilized BgpA in different proportions of various organic reagents was determined, and the results were shown in Fig. 5a. From the degree of influence of organic reagents on relative activity, the immobilized enzymes have an advantage in tolerating organic solvent over free BgpA. When the buffer contains 15% (*v/v*) organic solvent, methanol and DMSO have little effect on the relative activity of free and immobilized enzymes, while DES have a greater impact and decrease the activity quickly. When the buffer contains 60% (*v/v*) organic solvent, methanol has the greatest impact on relative activity among the solvents. As a matter of fact, the relative activity of immobilized enzymes does not significantly decrease at an organic reagent volume ratio of 15%. To further determine the capability of immobilized enzymes to tolerate organic reagents, they were incubated at different time intervals in 0–15% methanol, DES, and DMSO, and the changes in relative activity were calculated.

It was seen from Fig. 5b that the relative activity of both free and immobilized enzymes declined to varying degrees after prolonged incubation in buffers containing methanol. Especially, under the condition of 15% methanol, the relative activity was found to decline the fastest among all the concentration, and recent research suggests that methanol alters the molecular structure of proteins [38], thereby affecting enzyme catalytic activity. The moderate decline in the relative activity of immobilized enzyme was also observed, suggesting that the carrier was beneficial to protect the structure of BgpA.

As shown in Fig. 5c, an unexpected decrease in relative activity was observed after the free enzyme was incubated in buffer without organic solvent. Meanwhile after incubation in buffers composed of DMSO for 2 h, both the free and immobilized enzymes maintained relative activity at 100% or even above. Among them, in the buffer containing 15% DMSO, the relative activity of immobilized enzyme was much higher than that of free enzyme, indicating that immobilized enzyme can remain stable against 15% DMSO.

From Fig. 5d, it was seen that the relative enzyme activity of both free BgpA and R-SBA-15@BgpA in buffers containing DES were increased within the initial phase and then returned to around 100% when the incubation lasted for 2 h. It is particularly noteworthy that the relative enzyme activity of the free enzyme can reach as high as 300% in the buffer composed of 5% DES, followed by a rapid decline with prolonged incubation time. However, the relative activity of immobilized enzyme did not show an intensive increase or decrease, this is pertaining to the fact that DES are solvents enhancing solubility but of high viscosity and the higher solubility of substrates in the DES. Thus, for both free and immobilized enzymes, the increase in the solubility helps diffuse substrates and products, thereby accelerating contact between substrates and active sites and subsequently enhancing catalyst’s performance [39, 40]. However, the high viscosity could have greatly blocked the reaction between substrates and the immobilized enzyme, so no significant advantage of the immobilized enzyme over the free enzyme was observed when DES were incorporated into the buffer.

The results have confirmed the role of immobilization in improving BgpA resistance to organic solvents. Good organic solvent tolerance means that the organic groups offered by R-SBA-15 have created enzymes with a suitable microenvironment to prevent enzyme denaturation [41]. DES and DMSO, as co-solvents, were expected to play a helpful role in enzyme hydrolysis of natural products. The results suggested that DES were suitable for hydrolysis reactions using free enzymes, while DMSO was more suitable for hydrolysis reactions by immobilized enzymes.

### Leaching test and storage stability

**Fig. 6:**
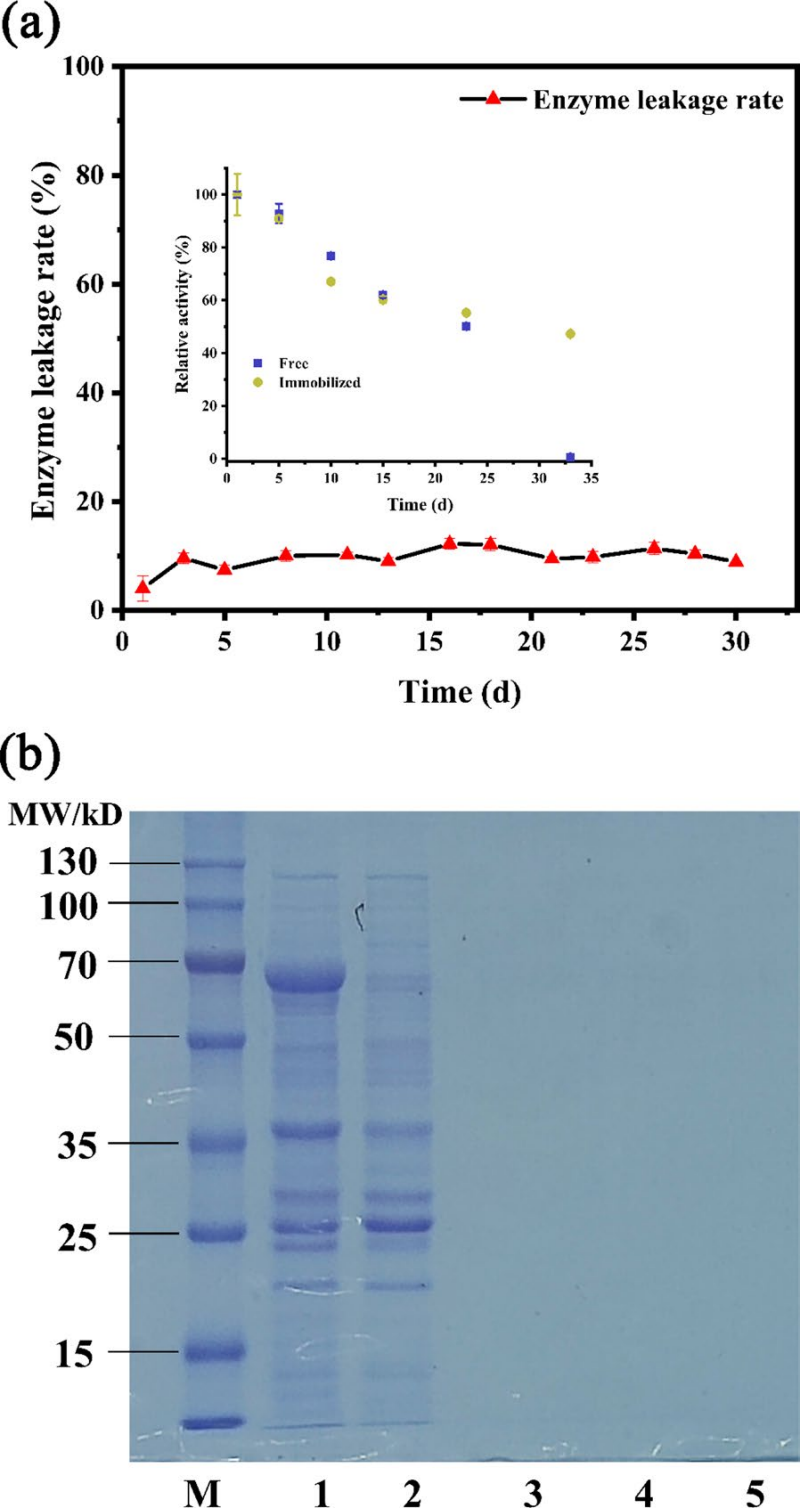
Leaching test and storage stability of R-SBA-15@BgpA. **a** leakage rate and storage stability of BgpA and R-SBA-15@BgpA. **b** SDS-PAGE profiles. Lane L. protein ladder; Lane 1. crude BgpA. Lane 2. unbound proteins of R-SBA-15; Lane 3. storage for 1 day. Lane 4. storage for 15 days. Lane 5. storage for 30 days. *BgpA* recombinant *β*-glucosidase from *Terrabacter ginsenosidimutans*, *R-SBA-15* N-aminoethyl-*γ*-aminopropyl trimethoxy group modified Santa Barbara Amorphous 15, *R-SBA-15@BgpA* immobilized BgpA on R-SBA-15

The results of the leaching test were shown in Fig. 6. From the SDS-PAGE results, the protein bands were not observed in the solution of immobilized enzyme on the 1st, 15th, and 30th day, indicating negligible enzyme leakage from R-SBA-15@BgpA. Gascón et al. [42] proposed that the limited amount of protein desorption was due to pore restrictions. Additionally, Yiu et al. [43] pointed out that the functional groups of modified SBA-15 prevent enzyme leaching, which was related to the interaction between the modified SBA-15 surface and the enzyme's functional groups. The firm ionic interaction between the enzyme and carrier prevents protein leaching. And, due to the presence of ionic interaction between carboxyl groups in BgpA and amine groups on the surface of R-SBA-15, the release rate of BgpA from SBA-15 functionalized by aminoethyl-γ-aminopropyl trimethoxy could be very limited.

The storage stability of both free and immobilized BgpA was determined, and the results were shown in Fig. 6. The immobilized enzyme R-SBA-15@BgpA retained over 50% of its relative activity even after continuous storage at 4 °C for 33 days. However, the relative activity of free BgpA almost lost, which could be due to the autolysis of BgpA in the phosphate buffer. The results confirmed the crucial role of immobilization in enhancing the storage stability of BgpA.

### Catalyst characterization

**Fig. 7:**
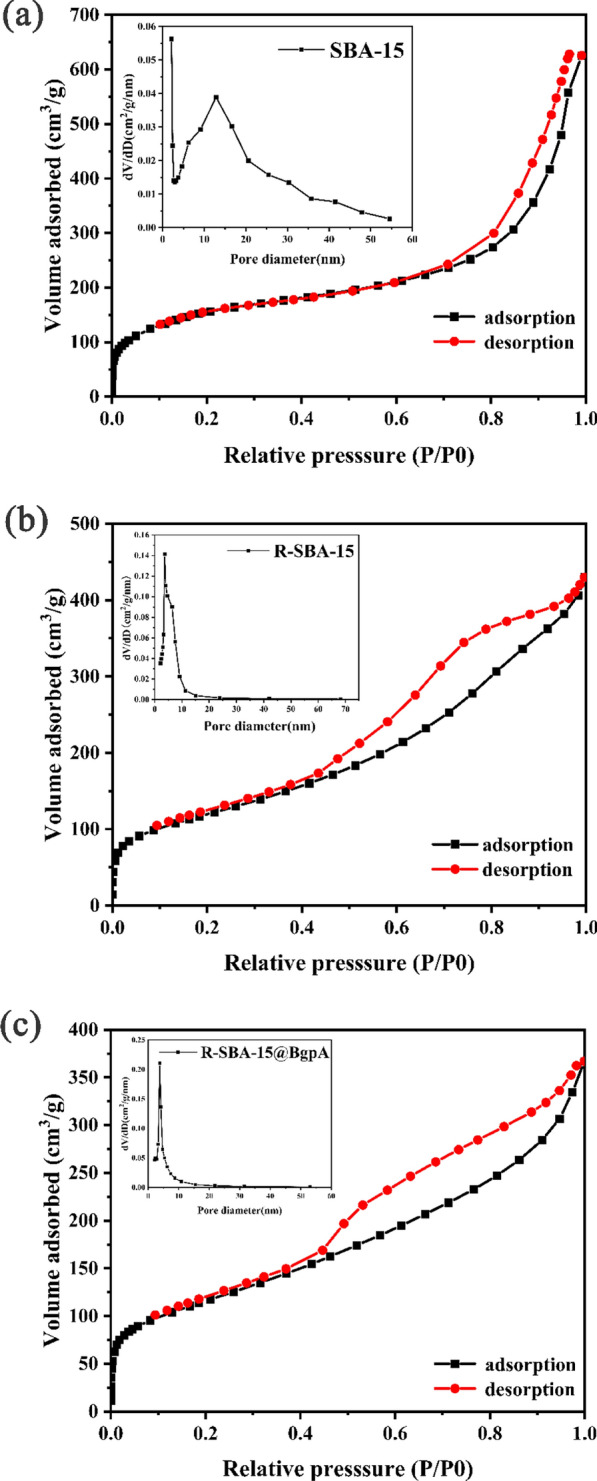
N_2_ adsorption–desorption isotherms and pore size distributions of the **a** SBA-15, **b** R-SBA-15 and **c** R-SBA-15@BgpA (BgpA: recombinant *β*-glucosidase from *Terrabacter ginsenosidimutans*; *SBA-15* Santa Barbara Amorphous 15, *R-SBA-15* N-aminoethyl-*γ*-aminopropyl trimethoxy modified SBA-15, *R-SBA-15@BgpA* immobilized BgpA on R-SBA-15

The N_2_ adsorption–desorption isotherms and pore size distribution of SBA-15, R-SBA-15, and R-SBA-15@BgpA were shown in Fig. 7. According to the IUPAC classification, all particles displayed typical IV-type isotherms with a well-defined H4-type hysteresis loop and sharp capillary condensation [36]. The H4-type hysteresis loop was a typical feature of ordered mesoporous materials with a two-dimensional hexagonal structure. Additionally, the shape of the hysteresis loop remained unchanged after organic modification and enzyme immobilization, indicating that the ordered mesoporous structure of SBA-15 remained intact. The pore size distributions of the parent material, modified SBA-15, and R-SBA-15@BgpA were also presented in Fig. 7 for comparison. The pore size, the pore volume and BET surface area of SBA-15 was 12.1 nm, 0.967 cm^3^/g and 568 m^2^/g, respectively. R-SBA-15 had pore size of 6.7 nm, BET surface area of 432 m^2^/g and pore volume of 0.665 cm^3^/g, and the resulting immobilized enzyme R-SBA-15@BgpA has a smaller pore size (6.5 nm), BET surface area (417 m^2^/g) and pore volume (0.568 cm^3^/g). As expected, the pore size, pore volume, and surface area of SBA-15 decreased after chemical modification. In particular, the incorporation of N-aminoethyl-*γ*-aminopropyl trimethoxysilane reduced the surface area of SBA-15, and the pore size distribution of R-SBA-15@BgpA indicated further reduction in pore size after successful enzyme immobilization in this application [44–46].

### Determination of epimedin A and sagittatoside A

**Fig. 8:**
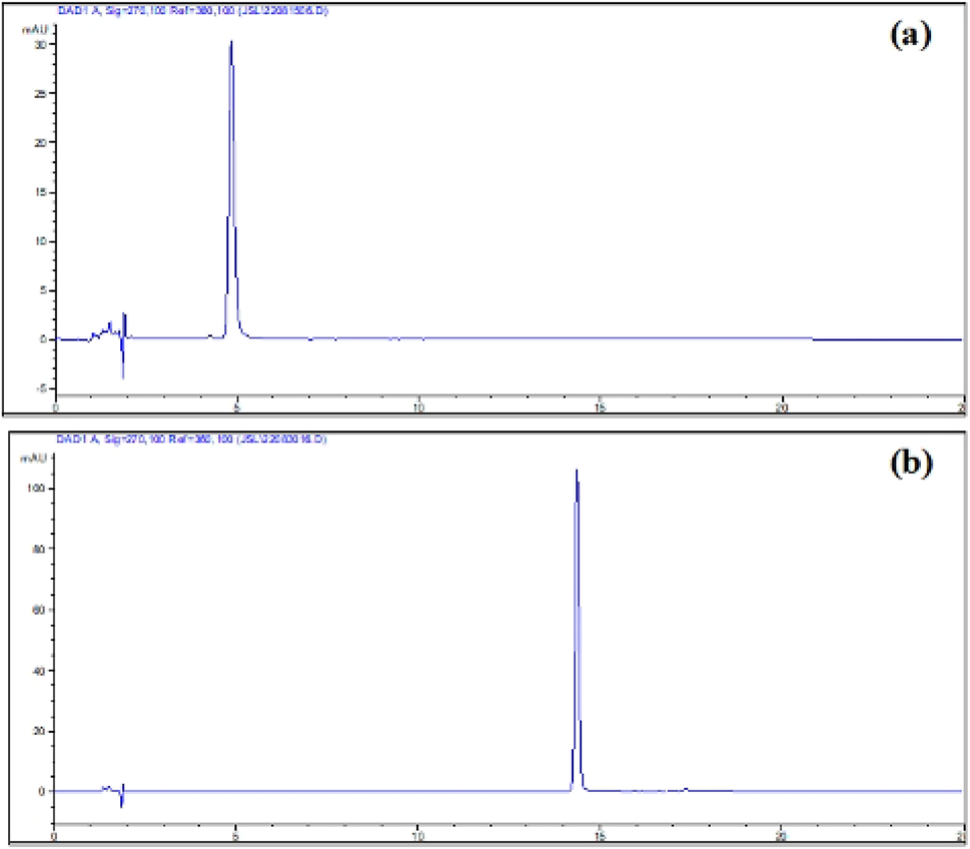
Typical HPLC–UV chromatograms of epimedin A (**a**) and sagittatoside A (**b**)

**Fig. 9:**
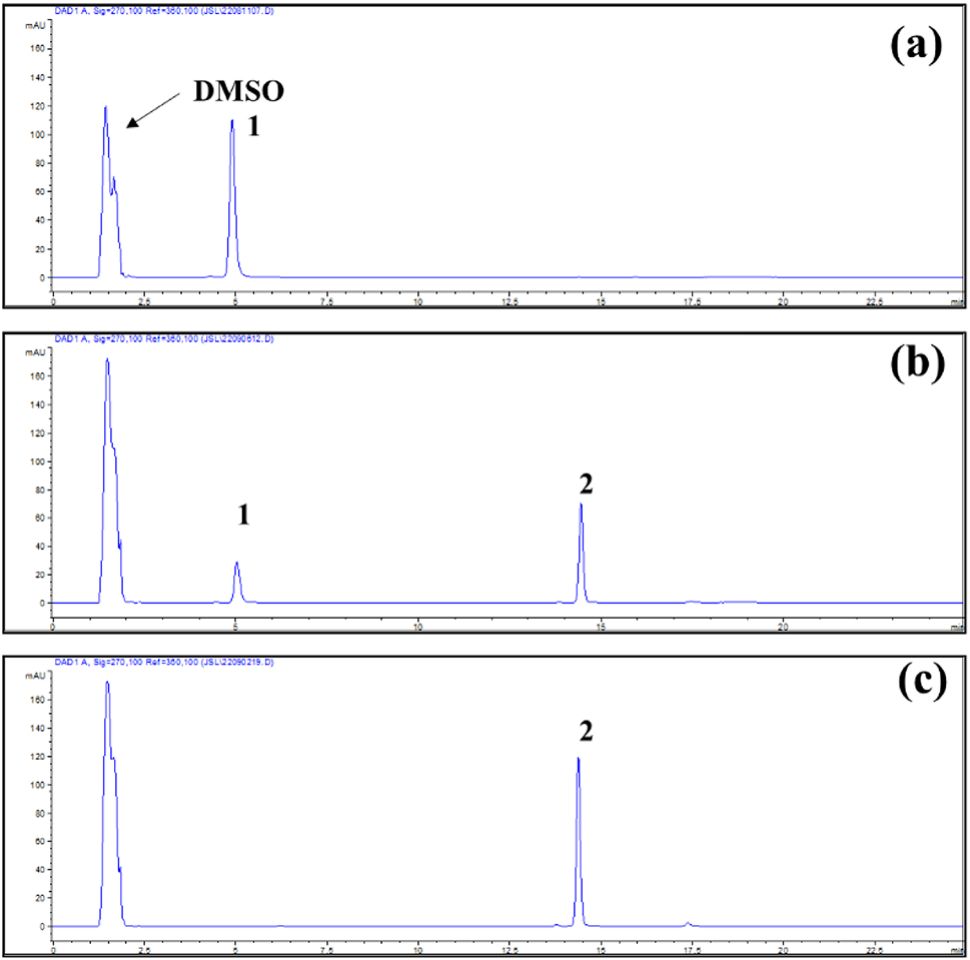
Typical HPLC–UV chromatograms of epimedin A (**a**); incomplete hydrolysis (**b**) and complete hydrolysis (**c**) (1: epimedin A, 2: sagittatoside A)

HPLC–UV chromatograms of epimedin A, sagittatoside A and sample solution were shown in Figs. 8 and 9. Standard curves of two references were drawn as Y = 5.776X + 16.8 (*r*^2^ = 0.9994) and Y = 5.7671X + 26.263 (*r*^2^ = 0.9994), separately, indicating good linear relationships within their concentration ranges.

### Conversion of epimedin A and catalyst reusability tests of R-SBA-15@BgpA

**Fig. 10:**
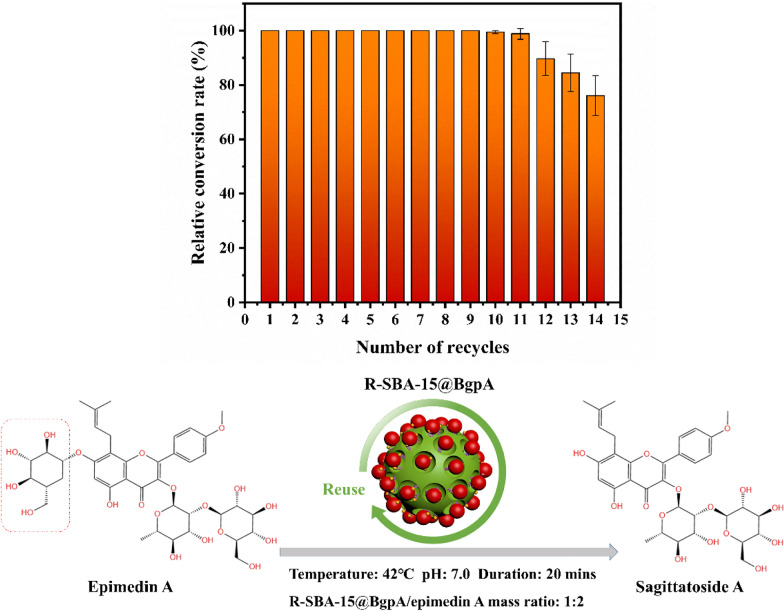
Reusability of R-SBA-15@BgpA for hydrolysis of epimedin A (R-SBA-15@BgpA: immobilized BgpA on N-aminoethyl-*γ*-aminopropyl trimethoxy modified Santa Barbara Amorphous 15)

For large-scale applications of an enzyme in industry, reusability is crucial in terms of efficiency and economy. Reusability of the R-SBA-15@BgpA was studied and results were presented in Fig. 10. Under the condition of 15% DMSO in phosphate buffer, the relative conversion rate of epimedin A was kept nearly 100% even after the R-SBA-15@BgpA has been used for ten cycles, and the R-SBA-15@BgpA showed an excellent stability against the reaction and remained 76.1% of its initial activity after fourteen cycles. It indicated that immobilized enzyme on R-SBA-15 were potential in practical application for preparation of natural products.

## Conclusions

In this study, SBA-15 with N-aminoethyl-*γ*-aminopropyl trimethoxy modification has been employed to immobilize BgpA, and the resulting R-SBA-15@BgpA showed a significant advantage with an activity recovery of 23.5%. Especially, the recombinant BgpA in the crude enzyme was completely captured by R-SBA-15 to achieve purification and immobilization of BgpA in one step. The immobilized enzyme R-SBA-15@BgpA has significantly improved thermal stability, organic solvent resistance, and storage stability compared to free BgpA. The performance in hydrolysis of epimedin A was evaluated for enzyme reusability, it was found that the R-SBA-15@BgpA showed an excellent stability against the reaction and remained almost 80% of its initial activity after fourteen cycles, it provided an ideal way for green production of sagittatoside A in pharmaceutical industry. In summary, functionalized SBA-15 could be a promising carrier for efficient purification and immobilization of a certain free enzyme from crude enzyme complex. In addition, R-SBA-15@BgpA was demonstrated to have high efficiency and stability, suggesting the great feasibility and potential to produce bioactive compounds such as secondary glycosides or aglycones from natural products.

## Experimental section

### Material and reagents

Mesoporous silica SBA-15 (L/N: 310002) was purchased from Nanjing XFNANO Materials Technology Co., Ltd. (China). Anilinomethyl triethoxysilane (≥ 95%, L/N: F1827134), N-[-3-(trimethoxysily) propyl) ethylenediamine (≥ 97%, L/N: B2215803) and P-nitrophenyl *β*-D-glucopyranoside (*p*-NPG, L/N: A2010058) were obtained from Aladdin Reagents Co., Ltd. (Shanghai, China). (3-Aminopropyl) triethoxysilane (≥ 99%, L/N: RH327270), Triethoxy (octyl) silane (≥ 97%, L/N: RH361523) and Trimethoxyphenylsilane (≥ 98%, L/N: RH322996) was provided by Shanghai Yi En Chemical Technology Co., Ltd. (China). Acetonitrile (ACN) of HPLC grade was supplied by Omni Gene LLC (US). Bicinchroninic Acid (BCA) Kit for protein assay, Coomassie Brilliant Blue R-250 and Isopropyl-*β-D*-thiogalactopyranoside (IPTG) were purchased from Sangon (Shanghai) Biotech Co., Ltd. (China). Protein ladder (15 kDa ~ 130 kDa) were purchased from Biosharp (Shanghai) Biotechnology Co., Ltd. (China). Epimedin A (purity ≥ 98.0% by HPLC–UV, L/N: 16123007) and Sagittatoside A (purity ≥ 98.0% by HPLC–UV, L/N: 20061501) were supplied by Chengdu Pufei De Biotech Co., Ltd. (China). Then the recombinant pGEX-BgpA was introduced into *E. coli*. by Jiangsu GenScript Biotechnology Co., Ltd. (Zhenjiang, China). All other solvents and reagents were of analytical grade and purchased from Sinopharm Chemical Reagent Co., Ltd. (China).

### Crude enzyme preparation

For expression of BgpA, *E. coli* containing pGEX-bgpA were first screened in LB-ampicillin solid medium and grown at 37 ℃ for 12 h, with single colonies picked into the liquid medium. The transformants harboring the plasmid were grown in LB-ampicillin medium at 37 ℃ until the cultures reached an OD of 0.6 at 600 nm, and protein expression was induced by 0.5 mM IPTG. The culture was further incubated at 20 ℃ for 6 h to express enzyme. Centrifugation was carried out at 5000 rpm for 5 min, and then proper amount of phosphate buffer (pH 7.0) was added to the resulting cell pellet. The mixture was subjected to sufficient homogenization under chilled condition at 1000 rpm in a high-speed homogenizer, prior to further centrifugation for crude enzyme solution [32].

### Protein profiling

Protein pattern of each fraction of interest was analyzed by 12% sodium dodecyl sulfate–polyacrylamide gel electrophoresis (SDS-PAGE), with a commercial protein ladder (15 *k*Da ~ 130 *k*Da) as the marker. Coomassie blue staining was performed for protein colorization, followed by visualization under daylight [47].

### Surface modification of SBA-15

Commercial SBA-15 particles were modified by organic functional groups through silanization. In details, 2.0 g of dried SBA-15 was dispersed in 60 mL of toluene, and 10 mmol of each silane coupling agent was added dropwise into the dispersion, which was kept refluxing under nitrogen atmosphere protection at 95 ºC for 8 h. The mixture was centrifuged at 8000 rpm for 20 min after the reaction, and the supernatant was discarded. Then the collected precipitate was washed with ethanol (50 mL) and ether (50 mL) respectively for three times, prior to drying at 52 ℃ for 24 h [36]. Eventually, SBA-15 had been functionalized by Anilinomethyl triethoxy, (3-aminopropyl) triethoxy, N-aminoethyl-*γ*-aminopropyl trimethoxy, N-octyl triethoxy and Trimethyloxy phenyl and they were named as X-SBA-15 (X was functional group).

### Immobilization of BgpA onto the carriers

A certain amount of crude enzyme solution was transferred into a conical flask, and then phosphate buffer (50 mM Na_2_HPO_4_-NaH_2_PO_4_, pH 7.0) was added to be 4.0 mL. SBA-15 or X-SBA-15 was suspended into the above solution, which was sonicated for 1 min to disperse them. Gentle shaking at 400 rpm was performed in a constant temperature incubator at 37 ℃ until the enzyme reached adsorption equilibrium for 30 min. Then, the suspension was centrifuged at 4000 rpm for 30 min and the collected pellet was washed with ultrapure water. The protein content in the supernatant was detected by BCA assay. The precipitate was freeze-dried to obtain immobilized enzyme SBA-15@BgpA or X-SBA-15@BgpA, and they were stored at 4 ℃ throughout the study.

### Determination of the amount of protein bound to carriers

Loading efficiency was calculated as the ratio of the amount of protein bound to the carrier to the initial amount of protein.

$${\text{Loading amount}}:\,{\text{L}} = \left( {C_{0} - C_{1} } \right)\, \times \,V/M_{s}$$

$${\text{Loading efficiency}}:\,{\text{E}}\left( {\text{\% }} \right) = (C_{0} - C_{1} )/C_{0} \, \times \,100\%$$

where *C*_*0*_ is protein concentration before immobilization, *C*_*1*_ is protein concentration after immobilization, *V* is the volume of enzyme solution, M_S_ is the weight of carriers.

### Enzymatic activity assay

The activities of the crude enzyme and its immobilized forms were determined using *p*NPG as substrate in 50 mM phosphate buffer (pH 7.0) at 37 °C. Reactions were stopped after 5 min by the addition of Na_2_CO_3_ solution, and the release of *p*-nitrophenol was measured immediately using a microplate reader at 405 nm (Thermo scientific; MULTISKAN FC). One unit (U) of BgpA activity was defined as the amount of protein required to generate 1.0 mmol of *p*-nitrophenol per min under the assay conditions. Specific activity was expressed as units per milligram of protein or its immobilized forms.

$${\text{Specific activity}}:\,{\text{U}}_{{\text{i}}} = C_{n} \, \times \,V\, \times \,N/(M_{t} \, \times \,t)$$

$${\text{Activity recovery}}:\,{\text{R}}\left( {\text{\% }} \right) = \,{{U_{i} \times M_{t} \times W_{f} } \mathord{\left/ {\vphantom {{U_{i} \times M_{t} \times W_{f} } {\left( {U_{f} \times L \times M_{s} } \right)}}} \right. \kern-0pt} {\left( {U_{f} \times L \times M_{s} } \right)}}\, \times \,100\%$$

where V is the total reaction system volume (mL), C_n_ is the concentration of *p*-nitro phenol (mmol), N is the dilution ratio of free or immobilized BgpA, M_t_ is the mass of free or immobilized BgpA (mg), *t* is the reaction duration, U_i_ represents the specific enzymatic activity of immobilized BgpA (*µ*mol·min^−1^·mg^−1^), U_f_ represents the specific enzymatic activity of free BgpA (*µ*mol·min^−1^·mg^−1^), L is the loading amount, M_s_ is the weight of carriers, W_f_ is the mass fraction of free enzyme in its immobilized forms. All data were acquired from the experiments in triplicate [9].

### Optimum pH and temperature of immobilized enzyme

The enzyme activity of R-SBA-15@BgpA particles were determined at different pH levels (3, 4, 5, 6, 7, 8 and 9). In brief, 20.0 mg of R-SBA-15@ BgpA was mixed into 2.0 mL of 0.05 M HAc-NaAc (pH3.0, pH4.0 or pH5.0), Na_2_HPO_4_-NaH_2_PO_4_ (pH6.0 or pH7.0) or Tris–HCl (pH8.0 or pH9.0). Meanwhile, the optimum pH for free BgpA was evaluated under the same reaction conditions. Relative enzymatic activity at each pH level was then calculated, which was expressed as a percentage relative to the maximum activity level. On the other hand, the enzyme activity of R-SBA-15@BgpA particles were measured at various temperatures. 20.0 mg of R-SBA-15@BgpA was added into 2.0 mL of 0.05 M Na_2_HPO_4_-NaH_2_PO_4_ (pH7.0). 25.0 μL of 1.0 mM pNPG was incubated with 25.0 μL of immobilized enzyme at 27 ℃, 32 ℃, 37 ℃, 42 ℃, 47 ℃, 52 ℃, 57 ℃, 62 ℃, 67 ℃, 72 ℃, 77 ℃ or 82 ℃ for 5 min. Meanwhile, the optimum temperature of free BgpA was also evaluated in a parallel condition. The temperature at which the enzymes exhibited their highest activity was considered as the optimum temperature, which was identified as 100% activity. The relative activity of the enzymes at each temperature was expressed as a percentage of the 100% activity.

### Thermal stability

The R-SBA-15@BgpA particles (20.0 mg) were incubated in 2.0 mL of 0.05 M Na_2_HPO_4_-NaH_2_PO_4_ (pH 7.0) at different temperatures (42 ℃, 47 ℃, 52 ℃, 57 ℃ and 62 ℃) for 6 h. Activity measurements were taken at different intervals to determine the thermal stability of the immobilized BgpA. In addition, the thermal stability of free BgpA was also evaluated under the same reaction. The initial enzyme activity was considered as 100%, and the relative activity was calculated accordingly.

### Organic solvent resistance

To investigate the stability of the immobilized enzyme in the presence of various organic solvents, 40.0 mg R-SBA-15@BgpA was incubated in 4.0 mL of 0.05 M Na_2_HPO_4_-NaH_2_PO_4_ (pH 7.0) containing different volume concentrations of organic solvents (15%, 30%, 60% of methanol, DMSO, or deep eutectic solvents) at 42 ℃ for 120 min. Deep eutectic solvent (DES) was composed of quaternary ammonium salt acting as a hydrogen bond acceptor (choline chloride, ChCl) and a hydrogen bond donor (ethylene glycol, EG), and the molar ratio of ChCl to EG was 1:2 (mol/mol). Afterwards, free BgpA and its immobilized forms were added to these solutions. The activity of each sample was measured by using the *p*NPG. Furthermore, free BgpA or R-SBA-15@BgpA particles were incubated in phosphate buffer (pH 7.0) containing 5% to 15% (*v/v*) methanol, DMSO, or DES at 42 ℃. Activity measurements were taken at different intervals (20 min) to determine the organic solvent resistance of the immobilized BgpA. The initial enzyme activity was considered as 100%, and the relative activity was calculated accordingly [48].

### Leaching test and storage stability

The stability of the interaction between BgpA and R-SBA-15 was evaluated at the leaching test. Briefly, 40 mg of the immobilized BgpA was dispersed in 4 mL of phosphate buffer (pH 7.0) and constantly incubated for 30 days. Aliquots were withdrawn at a planned time for the determination of protein content in the solution by BCA assay [49]. The immobilized enzyme solution and crude enzyme solution were stored at 4 °C for 33 days. The activity of each sample was measured against *p*NPG at different time span. The initial enzyme activity was considered as 100%, and the relative activity was calculated accordingly.

### Pore diameter, surface area measurement

N_2_ adsorption–desorption experiment was carried out on V-Sorb 2800TP surface area and pore size analyzer. Before the measurement, particles were degassed under vacuum at 100 ℃ for 8 h. The pore diameter was calculated on the adsorption isotherm using Barrett-Joyner-Halenda (BJH) method, and the surface area was calculated using Brunauer–Emmett–Teller (BET) method.

### HPLC–UV analysis of epimedin A and sagittatoside A

According to reported method with minor modification [34], the determination of epimedin A and sagittatoside A was conducted using an Agilent 1100 HPLC instrument equipped with a G1322A Degasser, G1312A BinPump, G1313A ALS Autosampler, G1316A Thermostatted Column Compartment and G1315A Photodiode Array Detector. The data and chromatograms were collected for processing via ChemStation Software (Rev.A.07.01[682]). All sample solutions were separated on a ZORBAX SB-C18 column (150 mm L. × 4.6 mm I.D., 5 μm). The column temperature was kept at 35°C throughout the analysis and the mobile phases consisted of ACN (A) and ultrapure water (B). Gradient elution was performed at a flow rate of 1.0 mL/min, and the time programs were as follows: 0 ~ 8 min, A (28%)—B (72%); 17 min, A (50%)—B (50%); 21 min, A (28%)—B (72%); and 21 ~ 25 min, A (28%)—B (72%). The UV detection wavelength was set at 270 nm and injection volume was 10 µL.

Epimedin A (10.0 mg) was precisely weighed and dissolved in MeOH and scaled to 25 mL as stock solution mixture (4.000 × 10^–1^ mg/mL). Then, a series of dilutions were performed to prepare standard solutions with concentrations at 2.000 × 10^–1^ mg/mL, 1.000 × 10^–1^ mg/mL, 5.000 × 10^–2^ mg/mL, 2.500 × 10^–2^ mg/mL, 1.250 × 10^–2^ mg/mL, 6.250 × 10^–3^ mg/mL and 3.125 × 10^–4^ mg/mL. In addition, sagittatoside A (10.0 mg) was precisely weighed and dissolved in MeOH and scaled to 10 mL as stock solution mixture (1.000 mg/mL). Then, a series of dilutions were performed to prepare standard solutions with concentrations at 5.000 × 10^–1^ mg/mL, 2.500 × 10^–1^ mg/mL, 1.250 × 10^–1^ mg/mL, 6.250 × 10^–2^ mg/mL, 3.125 × 10^–2^ mg/mL, 1.563 × 10^–2^ mg/mL, 7.813 × 10^–3^ mg/mL, 3.906 × 10^–3^ mg/mL, 1.953 × 10^–3^ mg/mL and 9.766 × 10^–4^ mg/mL.

Prior to any injection, all solutions were filtered through a 0.22 µm PTFE membrane syringe. To determine linearity, the standard solutions were analyzed and the peak area (Y) versus the concentration (X) of either epimedin A or sagittatoside A was then plotted for calibration curves by ordinary linear regression using Microsoft Office Excel 2003.

### Conversion of epimedin A and reusability tests of R-SBA-15@BgpA

To convert epimedin A by R-SBA-15@BgpA, 1.0 mL of 2.0 mg/mL epimedin A solution (phosphate buffer containing 15% DMSO) was mixed with 1.0 mL of 1.0 mg/mL immobilized BgpA in the buffer, and they were incubated at 42 ℃ for 20 min. The reaction mixture was centrifuged at 8000 rpm for 10 min and washed with the buffer, and the resulting precipitation was collected for the next cycle of conversion of epimedin A to prepare sagittatoside A. The initial conversion ratio was considered 100%, and the relative conversion ratio was calculated accordingly.

## Data Availability

The datasets generated during and/or analysed during the current study are available from the corresponding author on reasonable request.
